# Medium-term storage of platelet-derived orthobiologics: a feasible alternative for equine practice

**DOI:** 10.3389/fvets.2025.1720164

**Published:** 2026-01-12

**Authors:** Sarah Raphaela Torquato Seidel, Joice Fülber, Ângela Perrone Barbosa, Natalia Mori Avellaneda Penatti, Marilene Demasi, Raquel Yvonne Arantes Baccarin

**Affiliations:** 1 Departamento de Clínica Médica, Faculdade de Medicina Veterinária e Zootecnia, Universidade de São Paulo, São Paulo, Brazil; 2 Departamento de Cirurgia, Faculdade de Medicina Veterinária e Zootecnia, Universidade de São Paulo, São Paulo, Brazil; 3 Laboratório de Bioquímica, Instituto Butantan, São Paulo, Brazil

**Keywords:** freeze-dried PRP, orthobiologics, platelet lysate, platelet-rich plasma, regenerative medicine

## Abstract

**Introduction:**

Platelet-rich plasma (PRP) is commonly used by equine veterinarians. Although PRP is considered a feasible and affordable orthobiologic, its use has been associated with certain drawbacks, such as the time required for protocols and the necessity of using a fresh product, which is usually prepared at the time of use. In this context, the present study aimed to produce PRP-derived orthobiologics with the potential to be stored for longer periods while maintaining similar growth factor contents.

**Methods:**

PRP was prepared from six donors and subjected to additional processing: lysis, yielding Platelet Lysate (PL), and freeze-drying, yielding lyophilized PRP (FD-PRP). The three products were stored for 30 days at different temperatures (−80°C, −20°C, and room temperature [RT]). Additionally, orthobiologics from a single donor were also subjected to longer periods of storage: PRP, FD-PRP, and PL for 1 and 2 years at −80°C and −20°C; and FD-PRP at RT was stored for 1 year with cryoprotectant and 30 days without cryoprotectant.

**Results:**

Storage for 30 days presented distinct pattern related to the temperatures on each orthobiologic: there was no difference on TGF-β1 concentrations between the hemocomponents at −80°C, PL retained a high TGF-β1 concentration at −20°C, PRP and FD-PRP showed a slight increase in TGF-β1 content at RT when compared to PL. RT also resulted in decreases in IL-1β, IL-10, and TNF-α concentrations in all hemocomponents.

**Discussion:**

The protocols used in this study efficiently produced hemocomponents with similar content, offering some benefits, such as the possibility of preparing major volumes at the same time, preparing aliquots that would be ready to be used throughout the entire treatment, and storing them in farms, clinics, and hospitals.

## 1 Introduction

Platelet-rich plasma (PRP) is an orthobiologic agent widely used in equine regenerative medicine. Many studies have reported on its various applications, most of which are related to orthopedic conditions, such as articular diseases (1), tendinopathies (2), desmitis (3), and laminitis (4). Although some beneficial outcomes have been observed, high variability in obtention protocols and, consequently, in final products is a major pitfall jeopardizing the evaluation accuracy of this therapeutic potential for clinical intent (5, 6). Equally importantly, there is significant individual variability inherent to the use of different donors; the final product can have different compositions depending on whether it was collected over time from the same donor and using the same preparation methodology.

A brief literature review revealed a wide range of individual protocols and numerous commercially available kits. Although not every study reports on platelet and leukocyte counts, growth factors, and cytokine concentrations on these orthobiologics, it is important to consider that each hemocomponent could present a unique composition, not only related to the platelet content, but also to the active biomolecules that are primarily responsible for the therapeutic effects (7–9). It is also necessary to emphasize the time-consuming nature of manual protocols, which can take hours from collection to administration, as well as the difficulty of testing the sterility of fresh products before their application.

Platelet Lysate (PL) is obtained from platelet concentrates, such as PRP, through activation or rupture of the membrane, releasing biomolecules into the plasma (10); while lyophilization is a drying process intended to extend the preservation time of a given product, without loss of quality (11). Both procedures could result in orthobiologics with similar therapeutic characteristics to PRP, but with some advantages: the possibility of preparing major volumes at a single time and storing them, allowing the use of the same hemocomponent throughout the entire treatment, in addition to the potential to check the biosecurity/sterility before application.

Given these considerations, we hypothesized that it would be possible to obtain PRP-derived products with comparable biological characteristics that could be stored for extended periods, preferably at −20 °C, making them more practical and accessible for use in equine clinical settings. Therefore, this study aimed to evaluate the efficacy of lyophilization and lysis protocols for producing PRP-derived hemocomponents, and to assess the stability of these products under various storage conditions.

## 2 Methods

### 2.1 Animals

This study was approved by the Ethics Committee on Animal Use of the Faculty of Veterinary Medicine and Animal Science, University of São Paulo (CEUA/USP; 9,643,220,218). Six horses of different breeds (three male and three female), aged between 6 months and 8 years, were used as blood donors. All were free of systemic diseases and had no history of taking drugs that could interfere with platelet activity for at least 15 days prior to blood collection.

### 2.2 Hemocomponent production

**Figure 1:**
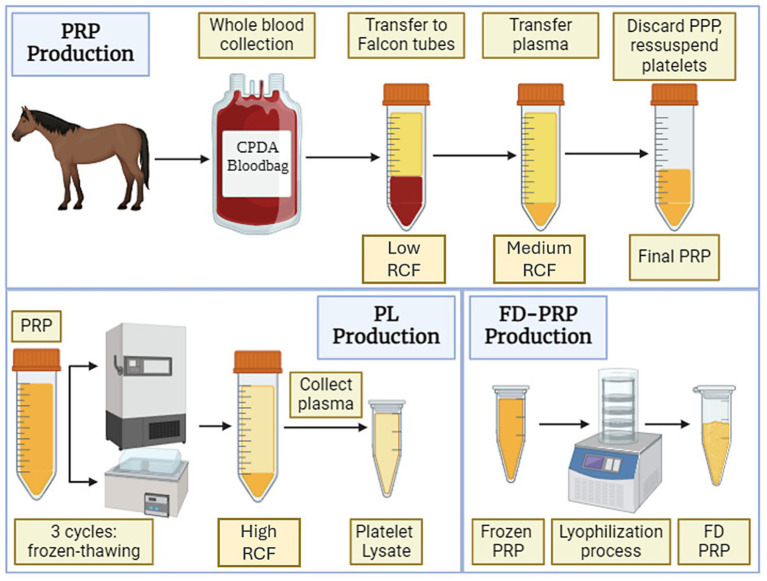
Schematic illustration of the protocols used to obtain hemocomponents. Platelet-poor plasma (PPP), platelet-rich plasma (PRP), platelet lysate (PL), freeze-dried PRP (FD-PRP). Images were generated using Biorender.com.

Blood was collected from the jugular vein after clipping and aseptic preparation. A blood bag (JP Farma, Ribeirão Preto, Brazil) containing CPDA as an anticoagulant (sodium citrate, sodium phosphate, dextrose, and adenine) was used for hemocomponent production in each animal, as well as an EDTA tube for basal cell counting. All the protocols used are summarized in Figure 1.

### 2.3 Platelet-rich plasma

Following collection, the blood bag was vertically positioned and allowed to sediment for 30 min, dividing it into two major layers: red cells at the bottom and plasma at the upper part. A validated protocol was used (12), with some adaptations necessary to avoid a platelet concentration greater than 6x as well as leukocyte enrichment. Plasma was carefully transferred to Falcon tubes (50 mL), and centrifuged at 350 × g for 7 min. Plasma was collected avoiding the collection of red cells or buffy coat, transferred to new Falcon tubes (50 mL), and centrifuged at 750 × g for 15 min. Subsequently, 70% of the plasma (only the upper part, classified as platelet-poor plasma [PPP]) was collected and discharged, and the remaining volume was homogenized to obtain platelet-rich plasma. Because PRP was used as the base hemocomponent for the production of all hemocomponents evaluated in this study, the final volume was divided into three parts: 1/3 for PRP, 1/3 for lyophilized PRP, and 1/3 for PL.

### 2.4 Lyophilized platelet-rich plasma

Lyophilized PRP (or Freeze-Dried PRP [FD-PRP]) was prepared according to a validated protocol for the lyophilization of human PRP (13), with some modifications. In brief, 140 μL of a cryoprotectant base was added for each 1,000 μL of fresh PRP. This stabilizing buffer consisted of equal parts of Tris base, glycine, and sucrose, and was added to PRP from all animals. To proceed with the lyophilization (FreeZone® Triad™ Freeze Dry System, Labconco, model 7,400,030 – Instituto de Ciências Biomédicas – ICB – USP), the fresh PRP (with and without cryoprotectant) was frozen at −80 °C at 45 degrees of inclination in 15 mL plastic tubes to increase the surface area of the hemocomponent. The lyophilizer was programed with the following settings: the platform temperature at −50 °C, the serpentine at −80 °C, and the collector temperature at −60 °C; with 1.03 mbar. The lyophilization process was performed with platform temperature at 0 °C, and 1.03 mbar, for aproximately 16 – 18 h. After the complete lyophilization, the tubes were closed and the samples were stored at −80 °C.

### 2.5 Platelet lysate

For PL production, fresh PRP was subjected to a freeze–thaw sequence to activate and disrupt all platelets, setting the free plasma content. Three cycles of complete freezing and thawing at −80 °C and 37 °C, respectively, followed by a centrifugation at 3,000 × g for 30 min, were conducted. All the plasma was collected, avoiding disturbance of the fragmented pellet on the bottom, transferred to new tubes, and stored at −80 °C.

### 2.6 Storage conditions

**Figure 2:**
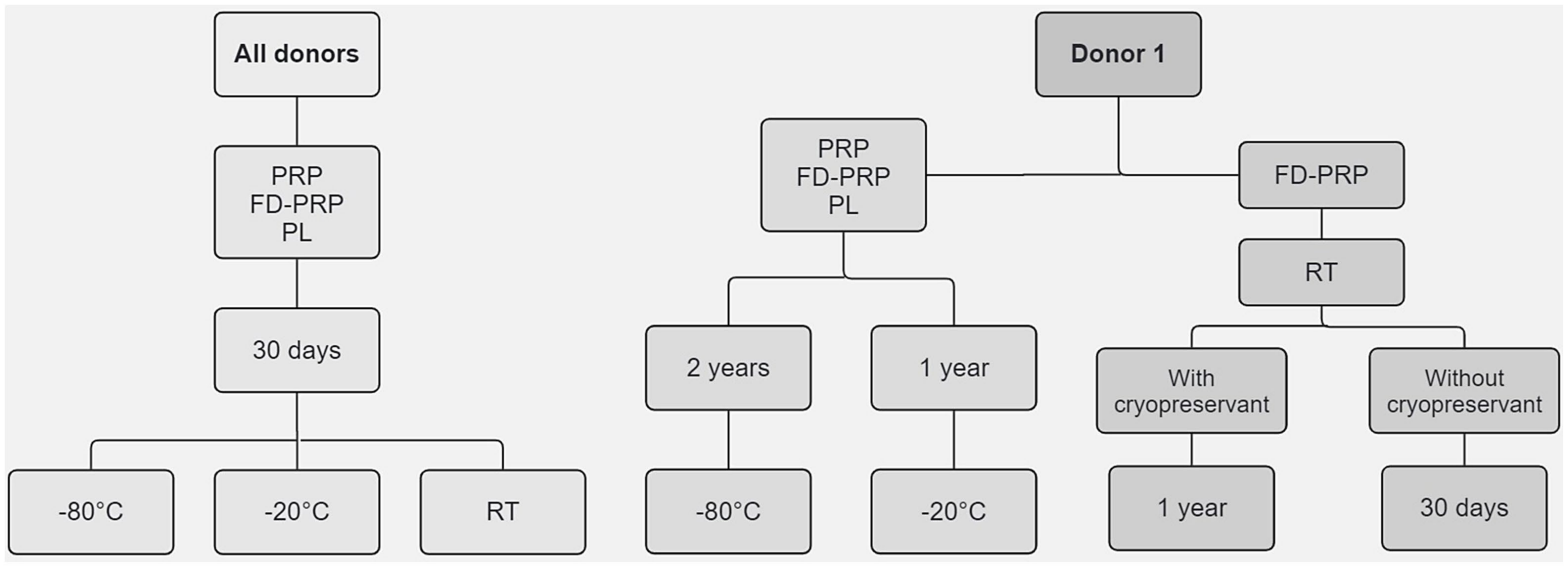
Flowchart of the storage conditions of the hemocomponents evaluated according to timeframe and temperature.

To evaluate the growth factor (GF) stability during 30 days of storage, hemocomponents were stored at −80 °C (ultra-freezer, UF), −20 °C (regular-freezer, RF), and 25 °C (room temperature, RT). To better understand the impact of storage duration on growth factor concentration, the hemocomponents of donor 1 were subjected to an extended storage period. Aliquots of PRP, FD-PRP, and PL were stored at −80 °C for 2 years and at −20 °C for 1 year. Additionally, to evaluate the necessity of cryoprotectant addition prior to lyophilization, FD-PRP aliquots were stored at RT for 30 days (without cryoprotectant) and 1 year (with cryoprotectant). All storage conditions are summarized in a flowchart in Figure 2.

### 2.7 Cellular content quantification

A complete blood count was performed for each blood sample collected from EDTA tubes. Red blood cells (RBC), leukocytes (white blood cells, WBC), and platelets (PLT) in the PRP samples were counted using a flow cytometry hematology system (ADVIA 2120i, Siemens, Erlangen, Germany).

### 2.8 Protein, albumin and fibrinogen quantification

Total protein and albumin contents were measured for all hemocomponents using an automatic biochemistry analyzer (Labmax 240 Premium, Labtest, Lagoa Santa, Brazil). Fibrinogen was quantified using an automatic coagulometer with laser emission (Clotimer, Clot – Sorocaba, Brazil) through a fibrinogen assay (Fibrinógeno, Wiener Lab, São Paulo, Brazil). Each sample was analyzed in duplicate.

### 2.9 Growth factor and cytokine quantification

TGF-β1 was quantified by enzyme linked immunosorbent assay (ELISA), using a commercially available human TGF-β1 kit (DuoSet® ELISA Human TGF-β1, R&D Systems – Minneapolis, USA), previously validated for use in equine samples (14, 15). The samples were diluted 10 fold and analyzed in duplicate, all the instructions for analyses were followed from the data sheet.

The cytokine contents of IL-1β, IL-6, IL-10, and TNF-*α* was measured through a bead-based multiplex assay (MILLIPLEX MAP Equine Cytokine/Chemokine Panel, EQCYTMAG-93 K, Millipore Corporation – Billerica, USA). Samples were tested at three different concentrations: undiluted, diluted five times, and diluted 10 times, in duplicate, in order to obtain a dillution curve, following a previous report from another study (16). The analysis were perfomed in undiluted samples in unicate.

Immunoblotting analyses were also performed to confirm the integrity of TGF-β1. Samples derived from donor 1 were selected for this purpose, stored at −80 °C for 2 years, −20 °C for 1 year and 30 days, and RT for 30 days. The final preparations (40 μg protein) were resuspended in SDS-PAGE sample buffer (60 mM Tris, pH 6.8, added of 25% glycerol, 2% SDS, 0.1% bromophenol blue and 1 mM DTT). Immunoblotting was performed using the ECL (Enhanced ChemiLuminescence) Western Blotting System (GE Healthcare) according to the manufacturer’s instructions, and the antibodies anti-TGF-β1 (Abcam, ab92486) and anti-rabbit with HRP-tail (GE Healthcare) were used as primary and secondary antibodies, respectively, both at 1:1000 dilution.

### 2.10 Statistical analyses

Initially, a descriptive analysis was performed to calculate the mean; standard deviation; and minimum, maximum, and median values of the quantitative variables stratified by temperature. Data normality was verified using the Shapiro–Wilk test. Mean comparisons among temperatures for the quantitative variables were conducted using ANOVA, followed by Tukey’s multiple comparison test for data with a symmetric distribution. For asymmetry, comparisons were made using a generalized linear model fit with gamma distribution, followed by the Wald multiple comparison test. For all the tests, a significance level of 5% or the corresponding *p*-value was set. All analyses were performed using SAS for Windows, version 9.4.

## 3 Results

### 3.1 Cellular content

The hematocrit (HCT), RBC, WBC, and PLT counts of all donors were within the normal range for the species. The PRP resulted in an average of 807 × 10^3^ platelets/μL, equivalent to an enrichment of 5.3x compared to baseline (PRP final counting divided by whole blood basal counting). Some leukocytes were present on PRP, but with no enrichment (0.4x), and some erythrocytes were almost undetectable (Table 1).

**Table 1:** Cell counts in whole blood and PRP at baseline and its relationship (final concentration).

Measures	Whole Blood			PRP			Final concentration		
	RBC (x10^6^/μL)	WBC (x10^3^/μL)	PLT (x10^3^/μL)	RBC (x10^6^/μL)	WBC (x10^3^/μL)	PLT (x10^3^/μL)	RBC	WBC	PLT
Mean	6.6	8.2	153	0.3	3.1	807	0	0.4	5.3
SD	0.99	1.65	24.75	0.23	0.67	72.59	0.03	0.12	0.60

RBC, Red Blood Cells; WBC, White Blood Cells; PLT, platelets; PRP, Platelet-Rich Plasma (PRP). Final concentration: ratio of PRP/Whole blood count.

### 3.2 Fibrinogen, protein and albumin measurement

**Figure 3:**
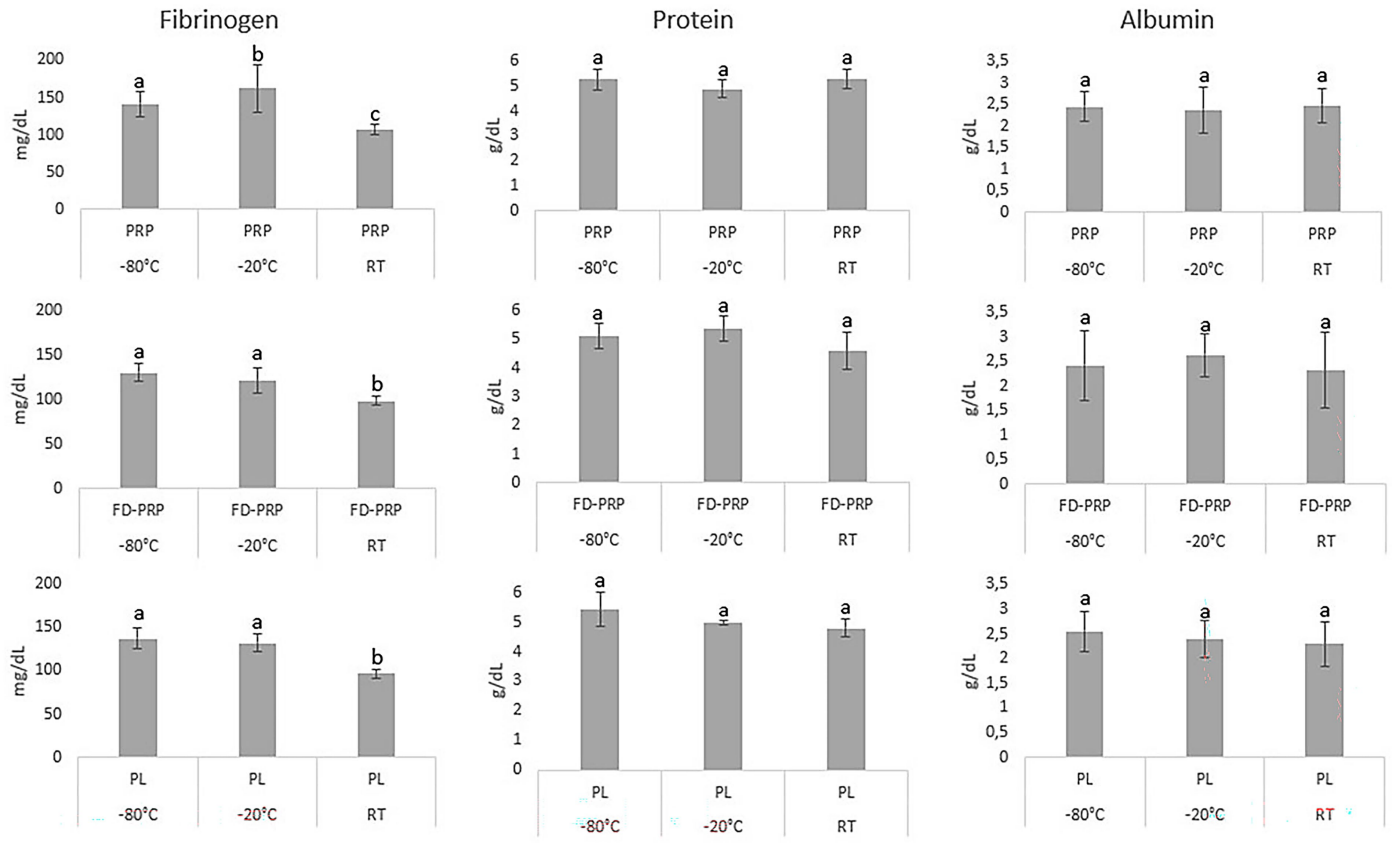
Fibrinogen, protein, and albumin concentrations in all hemocomponents stored for 30 days at different temperatures. Data for fibrinogen, protein, and albumin are asymmetrically distributed. Mean comparisons were made using gamma distribution and Wald’s test for multiple comparisons. Means followed by the same letter do not differ significantly at the 5% level.

The protein and albumin concentrations were similar for each hemocomponent, considering the different storage temperatures evaluated over a 30-day period. Fibrinogen was present in low concentrations in every hemocomponent stored at RT (Figure 3).

### 3.3 TGF-β1 quantification

**Figure 4:**
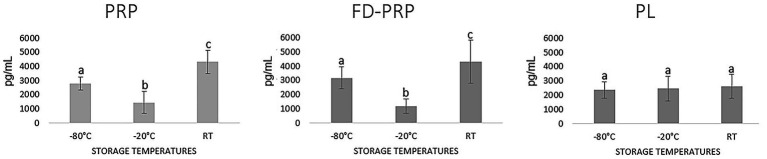
TGF-β1 concentrations on all hemocomponents stored for 30 days at different temperatures. Data from TGF-β1 showed a symmetric distribution. Mean comparisons were performed using analysis of variance and Tukey’s test for multiple comparisons. Means followed by the same letter do not differ significantly at the 5% level.

Quantification after 30 days of storage at different temperatures revealed higher TGF-β1 concentrations in PRP (2779.94 pg/mL) and FD-PRP (3165.95 pg/mL) when stored at RT, but lower TGF-β1 concentrations when stored at −20 °C. In contrast, PL showed no statistically significant differences across the evaluated temperatures (2,360.79 pg/mL at −80 °C, 2,464.82 pg/mL at −20 °C, and 2,632.20 pg/mL at RT), demonstrating a more consistent pattern (Figure 4).

Aliquots from donor 1 stored at −80 °C for 2 years maintained their concentrations of TGF-β1, while those stored for 1 year at −20 °C showed a major decrease, with FD-PRP reducing to 34%, PRP to 30%, and PL, which had the major reduction, presenting only 19% of its initial GF content (Table 2).

**Table 2:** TGF-β1 concentrations (pg/mL) on donor 1 hemocomponents for different times and with different storage temperatures.

Hemocomponents	2 years	1 year
	−80 °C	−20 °C
PRP	2,783.72	817.80
FD-PRP	3,716.00	1,300.00
PL	2,508.15	497.20

FD-PRP, lyophilized PRP; PL, platelet lysate; PRP, platelet rich plasma.

Exclusively for donor 1 FD-PRP stored for 1 year at RT showed an increase in GF content (5,547.70 pg/mL), when compared to that stored for 2 years at −80 °C (3,716.00 pg/mL), but it macroscopically exhibited a brownish color when compared to the yellowish color of the frozen lyophilized PRP. Further, there was no difference regarding the addition of cryopreservant from the same donor when evaluating the FD-PRP stored at −80 °C for 2 years (3,716.00 pg/mL) compared to that stored at RT for 30 days (4,021.90 pg/mL).

**Figure 5:**
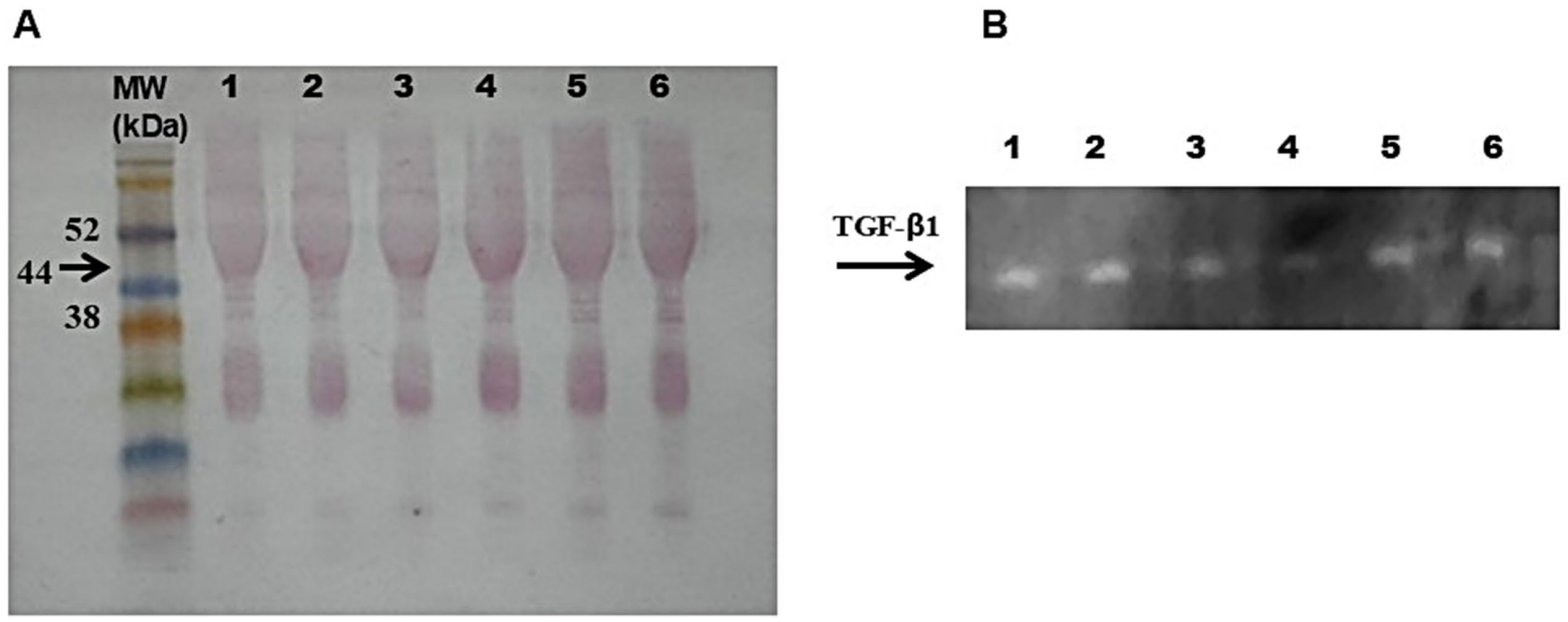
TGF-β1 identified on hemocomponents stored at −80 °C for 2 years and at RT for 30 days. **(A)** Membrane stained with Ponceau’s red, with the molecular weight (MW) standard run on the same gel. The arrow indicates the MW (44 kDa) of TGF-β1 protein. **(B)** Bands representing anti-TGF-β1 labeling are indicated by arrows. Samples: 1: FD-PRP -80 °C, 2: PL -80 °C, 3: PRP -80 °C, 4: FD-PRP RT, 5: PL RT, 6: PRP RT.

**Figure 6:**
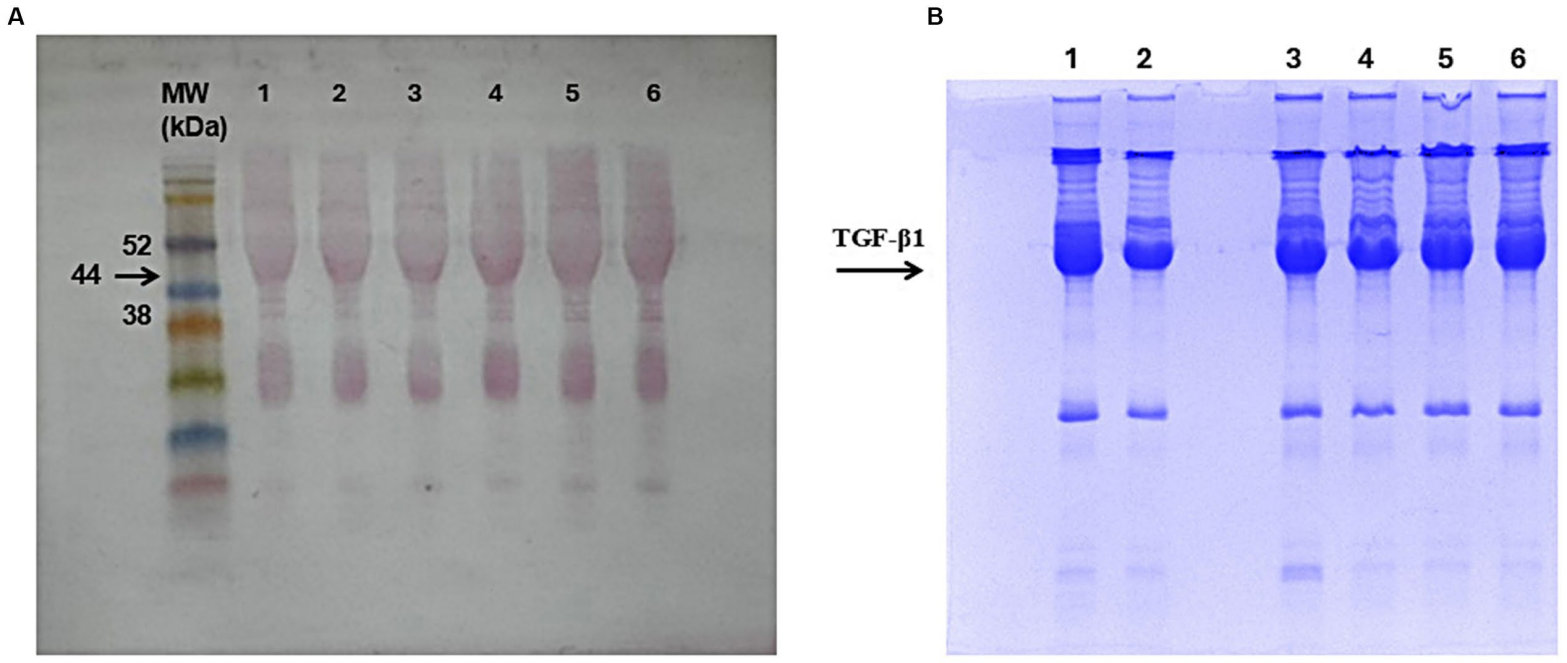
TGF-β1 identified on hemocomponents stored at −20 °C for 1 year and for 30 days. **(A)** Membrane stained with Ponceau’s red, with the molecular weight (MW) standard run on the same gel. The arrow indicates the MW (44 kDa) of TGF-β1 protein. **(B)** Polyacrylamide gel was stained with Coomassie brilliant blue. Samples: 1: FD-PRP 1 year, 2: PL 1 year, 3: PRP 1 year, 4: FD-PRP 30 days; 5: PL 30 days; and 6: PRP 30 days.

Results obtained from immunoblotting analyses highlighted no significant alteration of TGF-β1 preservation in samples stored at −80 °C for up to two years and RT for 30 days (Figure 5). TGF-β1 was also observed in samples stored at −20 °C for up to one year and 30 days (Figure 6A), with slight less intensity as observed on Coomassie Brilliant Blue staining in polyacrylamide gel (Figure 6B).

### 3.4 Cytokine quantification

Over the 30-day storage period, IL-6 was not detected in any of the samples at any of the temperatures evaluated. Further, IL-1β was detected only in samples from donor 2, with major concentrations on PRP (954.16 pg/mL) and FD-PRP (871.73 pg/mL) at −80 °C (nearly 10x when compared to PL [91.27 pg/mL]), a similar pattern at −20° with PRP (660.44 pg/mL) and FD-PRP (725.07 pg/mL) presenting higher concentrations (nearly 10x when compared to PL [51.11 pg/mL]). IL-1β presented similar concentrations in all hemocomponents at RT, which also presented the lowest values when compared to the previous temperatures: PRP (87.17 pg/mL), FD-PRP (72.3 pg/mL), and PL (76.45 pg/mL).

**Figure 7:**
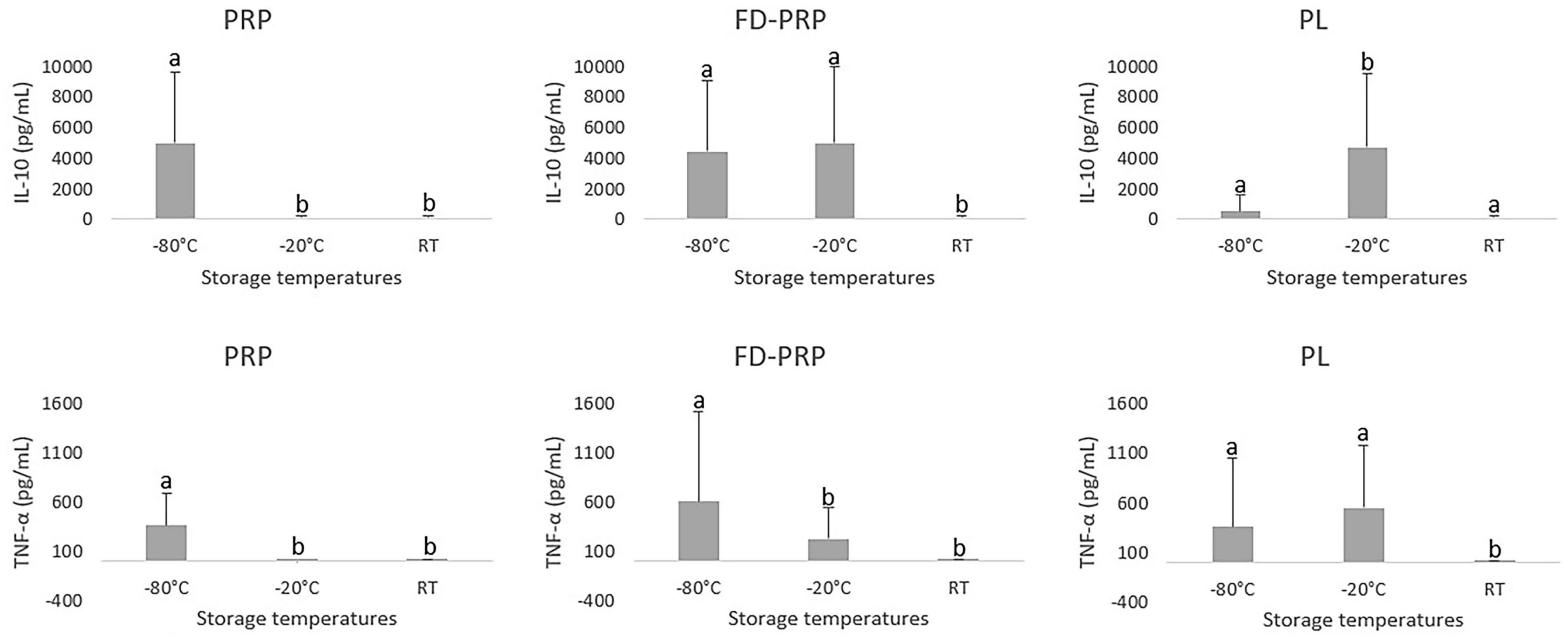
Cytokine concentrations in hemocomponents stored for 30 days at different temperatures. Data from IL-10 and TNF-*α* presented an asymmetric distribution. Mean comparisons were made using gamma distribution and Wald’s test for multiple comparisons. Means followed by the same letter do not differ significantly at the 5% level.

TNF-*α* revealed higher concentrations in FD-PRP at −80 °C and PL at −20 °C, being almost undetectable at RT (Figure 7).

IL-10 concentrations were the highest in PRP stored at −80 °C, followed by FD-PRP. At −20 °C, the highest concentrations were found in FD-PRP, followed by PL. In contrast, all hemocomponents stored at RT showed the lowest IL-10 levels (Figure 7).

Regarding the aliquots from donor 1 subjected to the long term storage, only TNF-*α* and IL-10 were detected. Samples stored at −80 °C for 2 years presented 587.8 pg/mL and 1392.73 pg/mL of TNF-α, and 3840.22 pg/mL and 2646.92 pg/mL of IL-10 in PRP and PL, respectively. None cytokine was detected in samples stored at −20 °C for 1 year.

## 4 Discussion

The protocol used to obtain PRP and its derivatives in this study proved to be feasible and reproducible, although it presented some peculiarities in the growth factor and cytokine contents observed among the final products depending on the storage conditions.

The cellular contents of the final PRP showed higher platelet enrichment, with a negligible presence of leukocytes and erythrocytes, compared to whole blood counting. Considering the importance of proper nomenclature, this hemocomponent should be classified as Leukocyte Platelet-Rich Plasma (L-PRP); although there was no leukocyte enrichment, WBCs were still present, with a low fibrin density (17). The final platelet concentration remains a subject of ongoing debate among authors, but even a two-fold concentration could be considered as PRP, as the final count is above the baseline (18). In our experience, platelet enrichment should not be used as a unique reference for PRP but also for the total number of platelets per milliliter, mainly considering that the final volume of therapeutic administration is calculated with reference to the capacity of local infiltration, avoiding unnecessary distension.

Regarding growth factor measurement, a similarity was observed among the hemocomponents, which is reasonable considering that a unique product was the main source for the other productions. It is also worth mentioning that TGF-β1 is an important growth factor related to the articular environment, being associated with the production and maintenance of cartilage extracellular matrix (ECM), presenting chondroprotection and anti-inflammatory action (19). The protocol chosen for PRP production was previously validated for equine species (12), and only minimally modified to achieve a maximum platelet concentration of 6x, avoiding the leukocyte enrichment and platelet activation throughout the processing. Considering the TGF-β1 concentration together with the platelet count, the GF values achieved were similar to those described for PRP activated by collagen or autologous thrombin (20, 21).

The similarity in TGF-β1 concentration detected on all hemocomponents evaluated for 30 days of storage at 80 °C indicates that the freeze-drying process preserved the platelet morphology and the response to release of the growth factors, and allowed the disruption of the platelet membrane on lysis processing in PL production, which resulted in the presence of these components in plasma. Furthermore, this similarity in GF concentration at −80 °C could be expected, as this temperature is considered the gold standard to maintain the majority of biological products for longer periods.

PL presented the same TGF-β1 concentration compared to PRP and FD-PRP at −80 °C, but concentrations were higher at −20 °C, and minor at RT. These results highlight the stability of this product, even at a domestic freezer temperature (−20 °C), which could be attributed to the absence or minimal presence of platelets, which were efficiently activated and disrupted during the lysis process, releasing their content in extracellular vesicles, with this lysis potentially improved by the freezing and thawing process (22). In general, a distinct process for obtaining platelet lysates results in products with unique compositions, including growth factors and extracellular vesicles (10). The variability observed on PRP and FD-PRP is potentially associated with the integrity of the platelets present in these products, which can continuously release GF at RT, but are not able to keep its stability and release at −20 °C.

The stability of TGF-β1 under different storage conditions has been demonstrated in several prior studies. For example, equine PL maintained its GF concentration for 8 days with major concentrations at 37 °C when compared to refrigerator temperature (23), and the supernatant released from equine PL gelled by the addition of CaCl further showed a steady concentration of this GF for 14 days at the same temperature (24).

Freezing fresh PRP is also considered a possibility to make this hemocomponent ready for use at any time. In a prior study, TGF-β1 presented the highest concentrations up to 6 months when stored at −20 °C, −80 °C and −196 °C compared to PDGF and IGF, which had the highest concentrations in fresh PRP activated with CaCl resuspended in lyophilized bovine thrombin (25). In another study, the authors concluded that the addition of DMSO or trehalose as a cryopreservant does not influence equine PRP, presenting similar TGF-β1 content regardless of the addition of these substances before the freezing process (26). This GF also exhibited a notable resistance to subsequent freeze–thaw cycles, and its concentration increased after a single freeze–thaw cycle (27).

To date, only a few studies have tested the lyophilization of equine PRP. Freeze-dried equine washed platelets resulted in similar TGF-β1 values when compared to fresh washed platelets, activated either with thrombin or platelet-activating factor (28). In another study, lyophilized PRP showed VEGF stability for at least 90 days, whereas PDGF decreased for the same time at room temperature (29). Both authors used trehalose as a cryopreservant, which enhances membrane integrity and reduces activation, maintaining the platelet functionality and response after processing, and extending platelet viability during storage (30). However, depending on the purpose, it may be necessary to remove the cryopreservant before application to minimize any possible adverse reactions, specially when using DMSO, the gold standard for platelet cryopreservation, but with toxicity properties (31). The use of deep eutectic solvents (DES), as the combination of L-proline and glycerol, have been considered for platelet cryopreservation with similar potential of DMSO, but with major biocompatibility (32). Furthermore, the addition of cryoprotectants to equine platelet lysates before freeze-drying process could have a positive impact on lyophilization process and on the final product presentation (33).

In the present study, the lyophilization process, along with the use of a cryoprotectant, yielded freeze-dried PRP with a growth factor content similar to that of the original PRP. In parallel, aliquots of freeze-dried PRP from one donor (donor 1), were tested with and without the addition of cryopreservant, while no difference in TGF-β1 concentrations were found between them, indicating that the addition of this substance could be dispensable for the 30 day storage period. This finding should be carefully interpreted, considering that ELISA methodology detect preserved epitopes and does not assess the biological activity of this molecules.

Concurrently, to achieve the major objective of this study, aliquots from a single donor (donor 1) were stored frozen for 2 years at −80 °C and 1 year at −20 °C. Subsequent analysis of these sample showed no variation of this GF content in samples when stored at −80 °C for 2 years; however, we observed a significant decrease between 60–80% in growth factor content in samples stored at −20 °C for 1 year, indicating that ultra-freezer temperatures are preferable for longer periods. Moreover, given that only samples derived from a single donor were subjected to extended periods of storage, further studies are necessary to substantiate this methodology for hemocomponents intended for long-term storage.

Room temperature is not a storage condition usually considered for longer storage of hemocomponents, and is commonly used to evaluate the stability of GF after PRP production (34). In fact, human PRP stored at RT showed turbidity, sedimentation, and a remarkable decrease in GF concentrations compared to lyophilized PRP, which was stable for up to 8 weeks under the same conditions (35). Surprisingly, we observed a slightly increase in TGF-β1 concentrations not only in FD-PRP, but also in PRP stored for up to 30 days at RT, when compared to those samples stored at −80 °C. This could be attributed to the membrane reorganization through processing, considering that freeze-thawing can result in larger EVs (36); also the RT could provide a more physiological environment to the platelets present in these hemocomponents, taking into account that some commercial orthobiologics are conditioned at 37 °C in order to obtain a major release of anti-inflammatory cytokines.

Another plausible explanation for the increase in GF observed in PRP and FD-PRP stored at RT may be TGF-β1 molecule fragmentation, which could be read as an integral molecule on ELISA methodology, whose integrity was verified by immunoblotting analysis. Nevertheless, although immunoblotting analysis confirmed the integrity of the TGF-β1 molecule on samples maintained at room temperature for 30 days, we do not encourage the storage of fresh PRP at RT for such a long time, particularly when it is subsequently used for therapeutic purposes. For longer storage periods at RT, FD-PRP should be the first choice, preferably hermetically vacuum-sealed, as is commonly done with lyophilized products made on an industrial scale to avoid oxidation and color alterations, as observed in our sample aliquoted in an Eppendorf tube.

Cytokine measurement is a valuable parameter that should be included for the proper characterization of orthobiologics. In the hemocomponents evaluated in our study, distinct concentrations were observed for the different storage temperatures, with major stability of IL-10 in frozen FD-PRP. IL-1β, an important pro-inflammatory cytokine, was detected only in hemocomponents from one donor (donor 2), reflecting the individuality factor on the final composition. Another pro-inflammatory cytokine, TNF-*α*, also showed major stability in frozen PL. Overall, we observed a significant decrease in the levels of cytokines stored at RT, suggesting a major sensitivity of their response to higher temperatures.

In human PRP, obtained from a protocol similar to that used in this study, the authors identified major detection of TNF-α on activated PRP, considering this a response to platelet stress induced by processing, centrifugation, and pipetting (7). IL-1β was positively correlated with neutrophils and monocytes in a leukocyte-Rich PRP (8), while in another study the authors did not find any correlation with platelet or leukocyte concentration, but observed higher concentrations of this interleukin in response to calcium activation (37). With regard to samples frozen at −80 °C and stored for 9 months, there were no changes in concentrations of IL-4, IL-10, and IL-13 on mechanically activated PRP (38).

Regarding the equine species, cytokine quantification has been predominantly conducted in the PL. Major concentrations of IL-1β and IL-10 were found on the third day in released PL gel, gradually diminishing on days 7 and 14 at 37 °C (24). Another study found a positive correlation between cytokines in platelet concentrates, including PL, and abnormal blood chemistry findings (16).

This study has some limitations. Firstly, only one growth factor presenting an important role related to articular tissues was measured; further assessment of other growth factors would provide more information about the stability of this product. In addition, we did not assess fresh PRP, as we froze our samples immediately after their preparation, which could interfere with platelet activation and the consequent release of growth factors. Similarly, we did not assess FD-PRP or PL immediately after their production. To perform this real-time evaluation, a different methodology and sample logistics should be used, considering the different finalization times of each product. Only aliquots from donor 1 were subjected to further analysis, including long-term storage and the comparison of using or not cryoprotectant agents, more studies are necessary to substantiate this results. Furthermore, it is important to emphasize that only quantitative immunological assays were used. These assays demonstrate molecular persistence but do not clarify the biological performance or actual regenerative potential of the final product. This includes recommendations for the use or not of cryoprotectants. Inclusion of functional assays, such as cell proliferation tests, would provide robust validation of the *in vivo* relevance of the results and should be considered in future studies.

## 5 Conclusion

In conclusion, the protocols used in this study all yielded similar concentrations of TGF-β1 among the different orthobiologics evaluated, regardless of the specific procedures inherent to each hemocomponent production. The storage temperature influenced both growth factor and cytokine content, with major concentrations of growth factors at room temperature and greater concentrations of cytokines on samples stored at −80 °C. Platelet lysate demonstrated greater stability in both analyses across all temperatures evaluated over the 30-day period, including at −20 °C, making it a more practical and accessible option for use in equine clinical settings. For longer periods, an ultrafreezer temperature is mandatory to preserve the PRP and PL content. Further, the addition of a cryopreservant could be not essential for maintaining the growth factor content of FD-PRP for 30 days of storage.

## Data Availability

The raw data supporting the conclusions of this article will be made available by the authors, without undue reservation.

## Supplementary material

SUPPLEMENTARY Image 1 Immunoblotting gel image.

SUPPLEMENTARY Image 2 Duplicate of Immunoblotting gel run.

SUPPLEMENTARY Image 3 Membrane stained with Ponceau’s red, with the molecular weight (MW) standard run on the same gel.

SUPPLEMENTARY Image 4 Polyacrylamide gel stained with Coomassie brilliant blue.

## References

[1] Garbin LC Olver CS. Platelet-rich products and their application to osteoarthritis. J Equine Vet Sci. (2020) 86:102820. doi: 10.1016/j.jevs.2019.102820, 32067662

[2] Geburek F Gaus M van Schie HTM Rohn K Stadler PM. Effect of intralesional platelet-rich plasma (PRP) treatment on clinical and ultrasonographic parameters in equine naturally occurring superficial digital flexor tendinopathies - a randomized prospective controlled clinical trial. BMC Vet Res. (2016) 12:191. doi: 10.1186/s12917-016-0826-1, 27604193 PMC5015224

[3] Romagnoli N Rinnovati R Ricciardi G Lambertini C Spinella G Spadari A. Clinical evaluation of intralesional injection of platelet-rich plasma for the treatment of proximal suspensory ligament Desmitis in horses. J Equine Vet Sci. (2015) 35:141–6. doi: 10.1016/j.jevs.2014.12.011

[4] Carmona JU Gómez WA López C. Could platelet-rich plasma be a clinical treatment for horses with laminitis? J Equine Vet Sci. (2018) 61:46–57. doi: 10.1016/j.jevs.2017.11.004

[5] Camargo Garbin L Lopez C Carmona JU. A critical overview of the use of platelet-rich plasma in equine medicine over the last decade. Front Vet Sci. (2021) 8:1–10. doi: 10.3389/fvets.2021.641818 PMC8044532 33869321

[6] Brossi PM Moreira JJ Machado TSL Baccarin RYA. Platelet-rich plasma in orthopedic therapy: a comparative systematic review of clinical and experimental data in equine and human musculoskeletal lesions. BMC Vet Res. (2015) 11:98. doi: 10.1186/s12917-015-0403-z, 25896610 PMC4449579

[7] Amable PR Carias RBV Teixeira MVT da Cruz PI do Amaral RJF C Granjeiro JM . Platelet-rich plasma preparation for regenerative medicine: optimization and quantification of cytokines and growth factors. Stem Cell Res Ther. (2013) 4:67. doi: 10.1186/scrt218 23759113 PMC3706762

[8] Sundman E a Cole BJ Fortier L a. Growth factor and catabolic cytokine concentrations are influenced by the cellular composition of platelet-rich plasma. Am J Sports Med. (2011) 39:2135–40. doi: 10.1177/0363546511417792, 21846925

[9] Boswell SG Cole BJ Sundman EA Karas V Fortier LA. Platelet-rich plasma: a milieu of bioactive factors. Arthroscopy. (2012) 28:429–39. doi: 10.1016/j.arthro.2011.10.018, 22284405

[10] Delila L Wu YW Nebie O Widyaningrum R Chou ML Devos D . Extensive characterization of the composition and functional activities of five preparations of human platelet lysates for dedicated clinical uses. Platelets. (2021) 32:259–72. doi: 10.1080/09537104.2020.1849603, 33245683

[11] Carpenter JF Chang BS Garzon-Rodriguez W Randolph TW. Rational design of stable lyophilized protein formulations: theory and practice. Pharm Biotechnol. (2002) 13:109–33. doi: 10.1007/978-1-4615-0557-0_5 11987749

[12] Seidel SRT Vendruscolo CP Moreira JJ Fülber J Ottaiano TF Oliva MLV . Does double centrifugation Lead to premature platelet aggregation and decreased TGF-β1 concentrations in equine platelet-rich plasma? Vet Sci. (2019) 6:68. doi: 10.3390/vetsci6030068, 31438534 PMC6789863

[13] Silva LQ de L MSA da S J-JA JLR CJ Huber SC Oliveira CC . Platelet-rich plasma lyophilization enables growth factor preservation and functionality when compared with fresh platelet-rich plasma. Regen Med. (2018) 13:775–84. doi: 10.2217/rme-2018-0035, 30284954

[14] Giraldo CE Álvarez ME Carmona JU. Effects of sodium citrate and acid citrate dextrose solutions on cell counts and growth factor release from equine pure-platelet rich plasma and pure-platelet rich gel. BMC Vet Res. (2015) 11:60. doi: 10.1186/s12917-015-0370-4, 25889052 PMC4364319

[15] Jiménez-Aristizabal RF López C Álvarez ME Giraldo C Prades M Carmona JU. Long-term cytokine and growth factor release from equine platelet-rich fibrin clots obtained with two different centrifugation protocols. Cytokine. (2017) 97:149–55. doi: 10.1016/j.cyto.2017.06.011, 28648869

[16] Moellerberndt J Hagen A Niebert S Büttner K Burk J. Cytokines in equine platelet lysate and related blood products. Front Vet Sci. (2023) 10:10. doi: 10.3389/fvets.2023.1117829, 36968472 PMC10033973

[17] Dohan Ehrenfest DM Andia I Zumstein MA Zhang C-Q Pinto NR Bielecki T. Classification of platelet concentrates (platelet-rich plasma-PRP, platelet-rich fibrin-PRF) for topical and infiltrative use in orthopedic and sports medicine: current consensus, clinical implications and perspectives. Muscles Ligaments Tendons J. (2014) 4:3–9. doi: 10.11138/mltj/2014.4.1.0013, 24932440 PMC4049647

[18] Fortier LA Hackett CH Cole BJ. The effects of platelet-rich plasma on cartilage: basic science and clinical application. Oper Tech Sports Med. (2011) 19:154–9. doi: 10.1053/j.otsm.2011.03.004

[19] Thielen NGM Van der Kraan PM Van Caam APM. TGFβ/BMP signaling pathway in cartilage homeostasis. Cells. (2019) 8:969. doi: 10.3390/cells8090969, 31450621 PMC6769927

[20] Textor JA Tablin F. Activation of equine platelet-rich plasma: comparison of methods and characterization of equine autologous thrombin. Vet Surg. (2012) 41:784–94. doi: 10.1111/j.1532-950X.2012.01016.x, 22742830

[21] Textor J Norris JW Tablin F. Effects of preparation method, shear force, and exposure to collagen on release of growth factors from equine platelet-rich plasma. Am J Vet Res. (2011) 72:271–8. doi: 10.2460/ajvr.72.2.271 21281204

[22] Troha K Vozel D Arko M Zavec AB Drago D Hocevar M . Autologous platelet and extracellular vesicle-rich plasma as therapeutic fluid: a review. Int J Mol Sci. (2023) 24:1–49. doi: 10.3390/ijms24043420 PMC9959846 36834843

[23] Camilo Osorio F Luís Ernesto Campos T Jéssica Guerra de O Henrique Carneiro L Leandro Abreu da F Andrés Ortega O . Comparative characterization between autologous serum and platelet lysate under different temperatures and storage times. Insights Vet Sci. (2023) 7:1038. doi: 10.29328/journal.ivs.1001038

[24] Naskou MC Tyma JF Gordon J Berezny A Kemelmakher H Richey AC . Equine platelet lysate gel: a matrix for mesenchymal stem cell delivery. Stem Cells Dev. (2022) 31:569–78. doi: 10.1089/scd.2022.0097, 35678071

[25] McClain AK McCarrel TM. The effect of four different freezing conditions and time in frozen storage on the concentration of commonly measured growth factors and enzymes in equine platelet-rich plasma over six months. BMC Vet Res. (2019) 15:1–9. doi: 10.1186/s12917-019-2040-4 31412868 PMC6694589

[26] Kwirant LA d A de la Corte FD Cantarelli C Cargnelutti JF Martins M Cabral MW . Cooling and cryopreservation of equine platelet-rich plasma with dimethyl sulfoxide and trehalose. J Equine Vet Sci. (2019) 72:112–6. doi: 10.1016/j.jevs.2018.10.009 30929774

[27] Fukuda K Kuroda T Tamura N Mita H Kasashima Y. Optimal activation methods for maximizing the concentrations of platelet-derived growth factor-bb and transforming growth factor-β1 in equine platelet-rich plasma. J Vet Med Sci. (2020) 82:1472–9. doi: 10.1292/jvms.20-0167, 32814750 PMC7653321

[28] Tablin F Walker NJ Hogle SE Pratt SM Norris JW. Assessment of platelet growth factors in supernatants from rehydrated freeze-dried equine platelets and their effects on fibroblasts in vitro. Am J Vet Res. (2008) 69:1512–9. doi: 10.2460/ajvr.69.11.1512, 18980435

[29] Freitas NPP Silva BDAP Bezerra MRL Pescini LYG Olinda RG Salgueiro CC d M . Freeze-dried platelet-rich plasma and stem cell-conditioned medium for therapeutic use in horses. J Equine Vet Sci. (2023) 121:104189. doi: 10.1016/j.jevs.2022.104189 36464033

[30] Gläfke C Akhoondi M Oldenhof H Sieme H Wolkers WF. Cryopreservation of platelets using trehalose: the role of membrane phase behavior during freezing. Biotechnol Prog. (2012) 28:1347–54. doi: 10.1002/btpr.1600, 22837111

[31] Read MS Reddick RL Bode AP Bellinger DA Nichols TC Taylor K . Preservation of hemostatic and structural properties of rehydrated lyophilized platelets: potential for long-term storage of dried platelets for transfusion. Proc Natl Acad Sci USA. (1995) 92:397–401. doi: 10.1073/pnas.92.2.397, 7831298 PMC42747

[32] Johnson L Bryant SJ Lei P Roan C Marks DC Bryant G. A deep eutectic solvent is an effective cryoprotective agent for platelets. Cryobiology. (2024) 116:104913–9. doi: 10.1016/j.cryobiol.2024.104913 38815783

[33] Bernardini C Romagnoli N Casalini I Turba ME Spadari A Forni M . Freeze-drying protocols and methods of maintaining the in-vitro biological activity of horse platelet lysate. Int J Vet Sci Med. (2024) 12:71–80. doi: 10.1080/23144599.2024.2380586, 39119550 PMC11308971

[34] Hauschild G Geburek F Gosheger G Eveslage M Serrano D Streitbürger A . Short term storage stability at room temperature of two different platelet-rich plasma preparations from equine donors and potential impact on growth factor concentrations. BMC Vet Res. (2016) 13:7. doi: 10.1186/s12917-016-0920-4, 28056978 PMC5216599

[35] Shiga Y Kubota G Orita S Inage K Kamoda H Yamashita M . Freeze-dried human platelet-rich plasma retains activation and growth factor expression after an eight-week preservation period. Asian Spine J. (2017) 11:329–36. doi: 10.4184/asj.2017.11.3.329, 28670400 PMC5481587

[36] Spakova T Janockova J Rosocha J. Characterization and therapeutic use of extracellular vesicles derived from platelets. Int J Mol Sci. (2021) 22:1–17. doi: 10.3390/ijms22189701, 34575865 PMC8468534

[37] Roh YH Kim W Park KU Oh JH. Cytokine-release kinetics of platelet-rich plasma according to various activation protocols. Bone Joint Res. (2016) 5:37–45. doi: 10.1302/2046-3758.52.2000540, 26862077 PMC4852788

[38] Brokhman I Galea AM. A novel method for the preparation and frozen storage of growth factors and cytokines obtained from platelet-rich plasma. J Cartil Jt Preserv. (2023) 3:100089. doi: 10.1016/j.jcjp.2022.100089,

