# Supplementary figures and images for: Medium-term storage of platelet-derived orthobiologics: a feasible alternative for equine practice
Source: Front Vet Sci. 2026 Jan 12;12:1720164. doi: 10.3389/fvets.2025.1720164 (PMC12832386; doi:10.3389/fvets.2025.1720164)

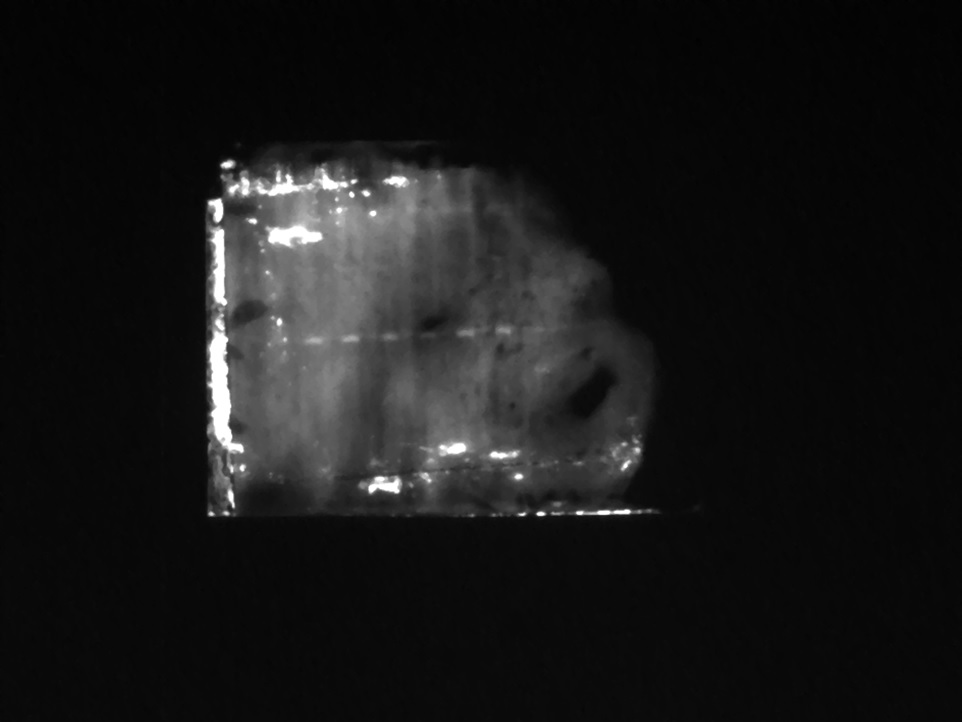

Supplement: SUPPLEMENTARY Image 1 — Immunoblotting gel image. [file Image_1.jpeg]

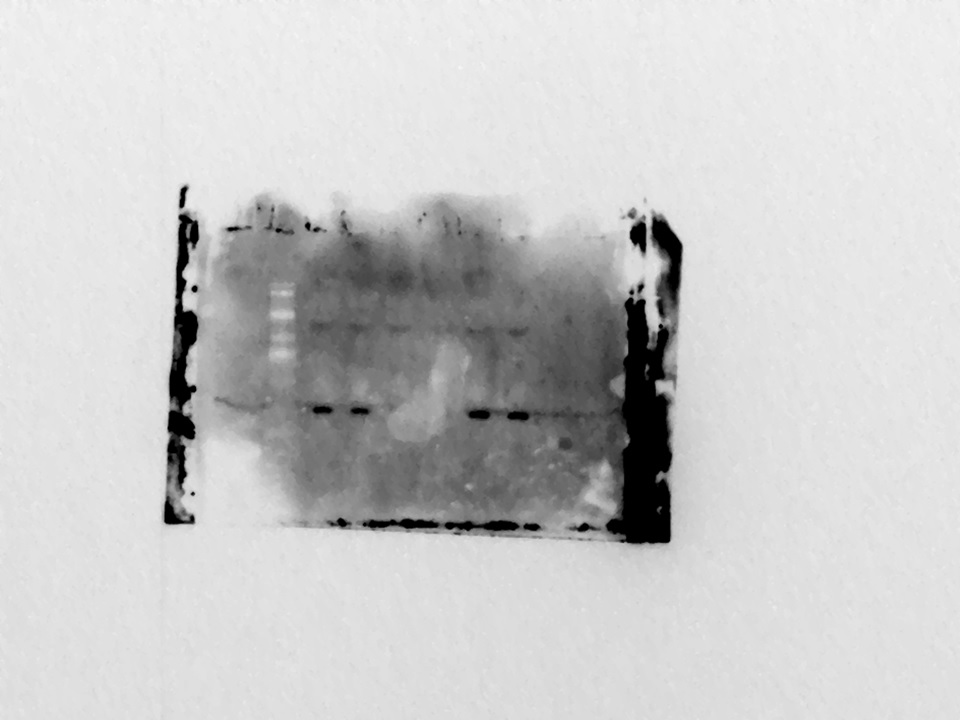

Supplement: SUPPLEMENTARY Image 2 — Duplicate of Immunoblotting gel run. [file Image_2.jpeg]

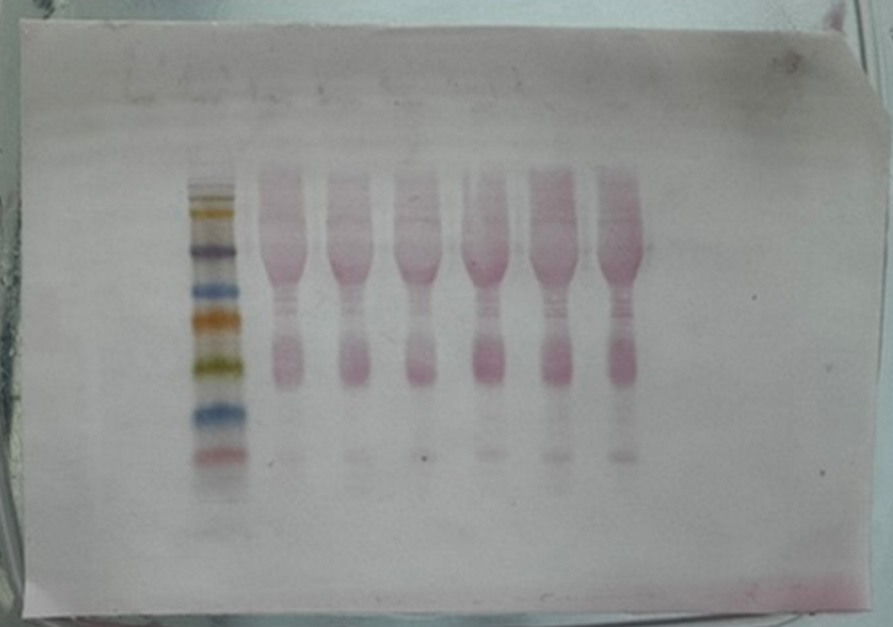

Supplement: SUPPLEMENTARY Image 3 — Membrane stained with Ponceau’s red, with the molecular weight (MW) standard run on the same gel. [file Image_3.jpeg]

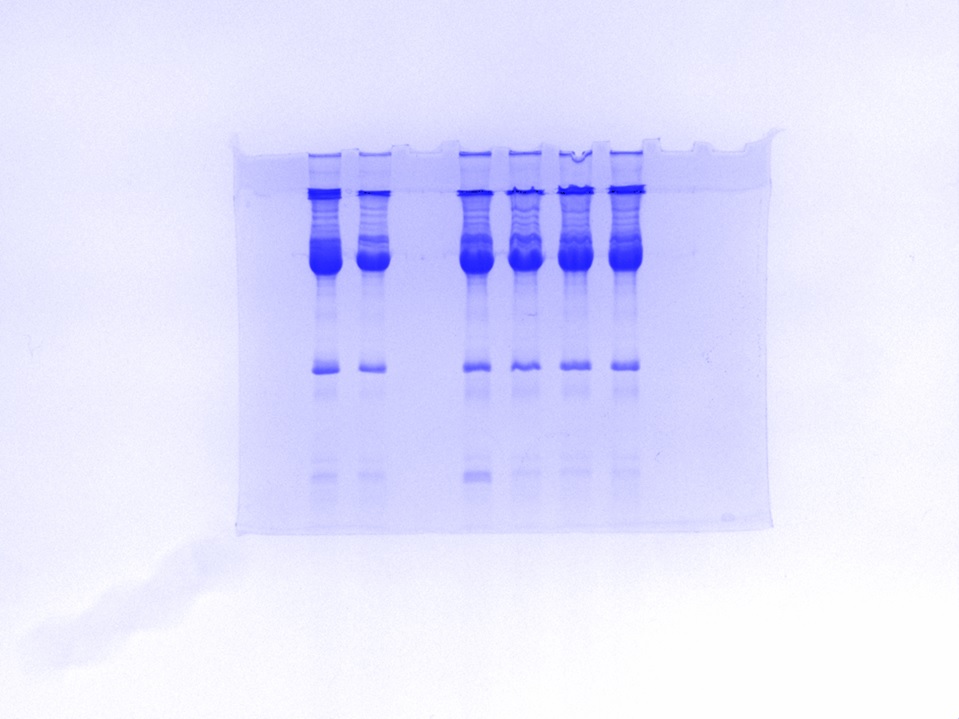

Supplement: SUPPLEMENTARY Image 4 — Polyacrylamide gel stained with Coomassie brilliant blue. [file Image_4.jpeg]
